# Contemporary Correction of Dentofacial Anomalies: A Clinical Assessment

**DOI:** 10.3390/dj4020011

**Published:** 2016-04-28

**Authors:** Nicolai Adolphs, Nicole Ernst, Erwin Keeve, Bodo Hoffmeister

**Affiliations:** Department of Craniomaxillofacial Surgery, Charité Universitätsmedizin Berlin, Campus Virchow-Klinikum, Augustenburger Platz 1, Mittelallee 2, Berlin 13353, Germany; nicole.ernst@charite.de (N.E.); erwin.keeve@charite.de (E.K.); bodo.hoffmeister@charite.de (B.H.)

**Keywords:** dentofacial deformities, computer-assisted cranio-maxillofacial surgery

## Abstract

Contemporary computer-assisted technologies can support the surgical team in the treatment of patients affected by dentofacial deformities. Based on own experiences of 350 patients that received orthognathic surgery by the same team from 2007 to 2015, this clinical review is intended to give an overview of the results and risks related to the surgical correction of dentofacial anomalies. Different clinical and technological innovations that can contribute to improve the planning and transfer of corrective dentofacial surgery are discussed as well. However, despite the presence of modern technologies, a patient-specific approach and solid craftsmanship remain the key factors in this elective surgery.

## 1. Introduction

Malocclusion can be differentiated into mild (dentoalveolar) or severe forms (skeletal), which may have an impact on facial appearance. If this is the case, the term “dentofacial anomaly” is typically used. Apart from functional impairment (mastication, breathing, speech, occlusal trauma with attrition, periodontal deterioration, affection of the temporomandibular joints), dentofacial anomalies may cause relevant psychosocial strain in affected patients. Mild-to-moderate forms of malocclusion may be corrected by orthodontic treatment alone. However the correction of severe types of malocclusion with an impairment of jaw relation typically requires surgical procedures to the lower and/or upper jaw in combination with orthodontic treatment. The surgical correction of dentofacial anomalies (“orthognathic surgery”) is one of the main domains in maxillofacial surgery and can contribute to reduce functional and psychosocial impairments in patients affected by dentofacial anomalies, resulting in a relevant improvement of quality of life [1]. Typically, this kind of elective surgery is performed after skeletal maturity within an interdisciplinary orthodontic and maxillofacial team approach [2,3,4]. Individual surgical correction of dentofacial anomalies may require three-dimensional reorientation of the jaws (“roll, pitch, yaw” [5]). The correction of dentofacial anomalies should address both physiologic occlusal situations with the correct relation of upper and lower jaw as well as facial balance and harmony by establishing symmetric facial proportions [6]. For that purpose, additional surgical procedures such as rhinoplasty or soft tissue corrections may be required and should be performed after the skeletal corrections.

Basic principles and techniques for the correction of dentofacial anomalies have been developed during the last century [7]. After the millennium, due to the ongoing “digitalization” in all fields of surgery, scientific efforts increasingly focused on improving the precision of planning and transfer of corrective orthognathic procedures. Computer-assisted technologies (imaging, virtual planning, rapid prototyping) and different developments and advances (piezosurgery, new implant designs) have contributed to ameliorate surgical service to patients who are affected by dentofacial anomalies and can also be applied to all levels of the craniofacial framework [8,9,10,11,12]. Based on personal clinical experience of one major center, this publication is intended to give an overview of current trends and concepts in this exciting surgical field.

## 2. Materials & Methods

Since 2007, 350 patients affected by dentofacial anomalies have been operated by the same surgical team within an interdisciplinary surgical-orthodontic approach at the Campus Virchow Klinikum, Charité Universitätsmedizin Berlin, Germany. The interdisciplinary orthognathic treatment planning focused on an optimal individual outcome regarding correct occlusal situations, as well as facial balance and harmony. Surgical steps and procedures were selected accordingly in order to provide reliable clinical results at a low risk. During that time, different innovations have been implemented in the daily clinical routine

Standard planning was based on clinical examination, standardized photo documentation, lateral cephalograms and orthopantomograms. Beginning in 2008, 3D-planning was available after the introduction of a cone beam CT (Iluma VisionDental^®^, IMTEC Europe, 61440 Oberursel, Germany). In selected cases, these datasets were also used for the computer-assisted fabrication of patient-specific models and/or corresponding wafers [13,14]. If a wafer was required, it was typically manufactured by a dental technician. However, if the preoperative orthodontic treatment was sufficient and postoperatively there were stable occlusal relations, no wafers were inserted. In cases of mandibulomaxillary corrections (“two-jaw surgery”), the planning and transfer of the procedure was performed in accordance to Reyneke’s concept of the “rotation of the occlusal plane” [15]. Control of the vertical relation was performed according to Kretschmer’s method [16].

Internal fixation was performed by using a semirigid system (Modus Oss 2.0, Medartis, Basel, Switzerland), which has originally been described by Kleier and co-authors. This system allows for the postoperative settling of the condylar processes in the glenoid fossae, and consequently no positional devices were required during the surgical procedures [17]. The material removal was typically performed after ending of the final orthodontic treatment; within this setting, the final clinical evaluation of the patients was performed by the surgical team with regard to complications and patient satisfaction.

From 2007, transpalatal distraction (TPD) has been implemented as the first surgical step for the correction of relevant transverse maxillary deficiency, replacing the preceding maxillary segmentation procedures with additional bone grafting. The initial maxillary widening by TPD was followed by orthodontic closure of the diastema and subsequent alignment, nivelation and decompensation of the dental arches. The surgical correction of the jaw relation was subsequently performed. Maxillary segmentation procedures were only performed in patients with minor transversal discrepancies. Resorbable plates and pins (Sonic Weld Rx^®^ System, KLS Martin, Tuttlingen, Germany), as well as piezosurgical devices, were available from 2007 on, however both technologies had only limited use with regard to orthognathic corrections (Figure 2c). Beginning in 2011, a series of skeletal class III patients were treated according to the concept of de Clerck before skeletal maturity through the use of skeletal anchorage and class III elastics (Figure 3a–h) [18].

## 3. Results

From January 2007 until May 2015, the same surgical team (one specialist, one resident) took care of the subspecialty of “orthognathic surgery” at Campus Virchow-Klinikum (CVK) being responsible for interdisciplinary planning with cooperating orthodontists, surgeries, postoperative care, follow-up and material removal. During that time, 350 corrections were performed in general anesthesia under stationary conditions. The distribution and type of surgeries over time are displayed in Table 1. Classic osteotomies of the upper and lower jaw are distributed quite equally. Noticeable is the high amount of distraction procedures, which is caused by an increasing acceptance of the transpalatal distractions (*n* = 78) within the group of cooperating orthodontists. The column “Other” integrates different procedures (segmental osteotomies, insertion of skeletal anchorage) which cannot be summarized in the classic osteotomies. Their peak in 2012 is mainly caused by the first insertions of elements for skeletal anchorage.

Final clinical evaluation of these patients at time of material removal allows the following statement with regard to therapeutic success, patient satisfaction and negative side effects after orthognathic surgery.

Minor complications (loosening of plates and screws with local inflammation, early partial recurrence of the initial malocclusion type within the first six months after surgery) were documented in each osteotomy group and could be managed without major drawback for the patient (early material removal, secondary correction). Relevant complications were extremely rare. In one patient undergoing “two-jaw surgery” massive bleeding requiring substitution of blood products occurred from retromaxillary venous plexus. One transpalatal distraction device failed due to infection and consecutive loosening in one patient with limited compliance. In two female patients bony consolidation failed to appear after LeFort I osteotomy. One of them suffered from severe bruxism, the other one became pregnant during the first six months after skeletal correction. Hormonal influence is likely to have contributed to that fact. In both patients secondary correction with additional bone grafting was subsequently required. No case of osteomyelitis or osteonecrosis of the jaws occurred.

With regard to neurosensory side effects after orthognathic correction, no reliable statement was possible. In the initial phase after surgery, most of the patients complained about reduced perception in the supply area of the corresponding nerves. At the time of material removal, this initial disturbance was regressive in the majority of patients. Permanent dysesthesia of the infraorbital nerve was not an issue. The complete permanent loss of neurosensory function of the inferior alveolar nerve has not been documented in these 350 patients, however, long-lasting dysesthesia in the chin region occurred regularly in patients undergoing mandibular advancement. Whether this neurosensory deficit is predominantly caused by the surgical split or to the traction on the neurovascular bundle cannot be differentiated. Overall, the incidence of well-known side effects of orthognathic surgical procedures was low in this group of 350 patients. Detailed preoperative education of the patient regarding typical and sometimes unavoidable complications, solid craftsmanship, as well as steady postoperative care by the operating team are certainly key factors for the successful surgical management of patients affected by dentofacial anomalies. Although the vast majority of patients indicated that they were satisfied with the result after orthognathic correction, there was a minority of patients who were not, although planning and transfer had been performed correctly. The management of this small group of patients may subsequently become challenging. Unrealistic preoperative expectations may be an indicator for such conditions.

Contemporary options for the interdisciplinary management of dentofacial deformities are demonstrated by four different representative patient cases.

### Case 1 (Figure 1a–l)

**Figure 1:**
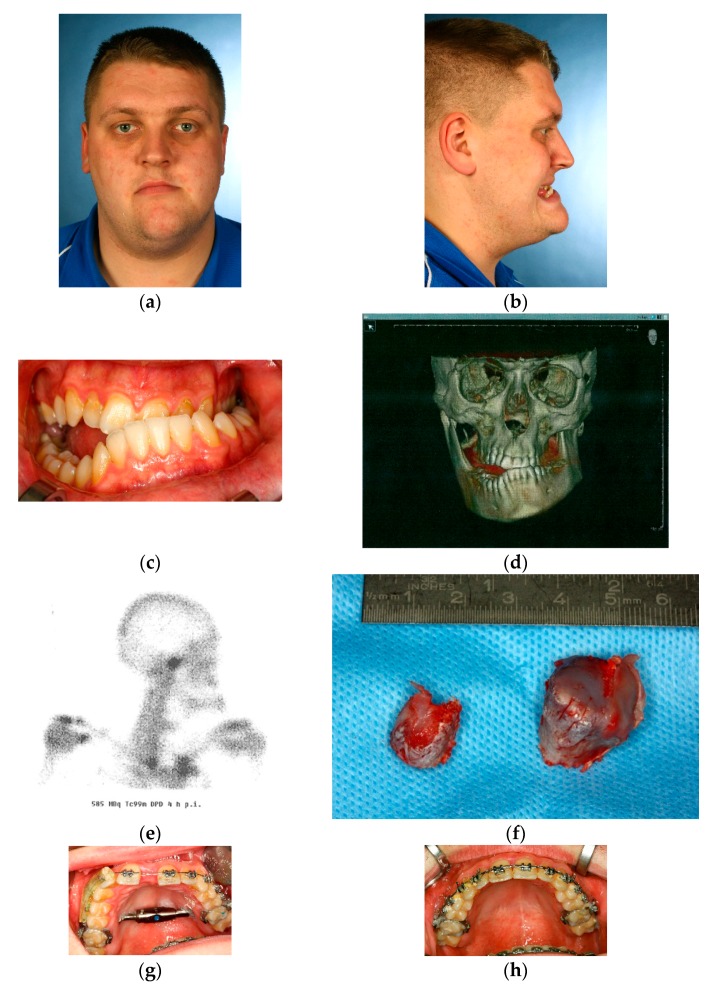

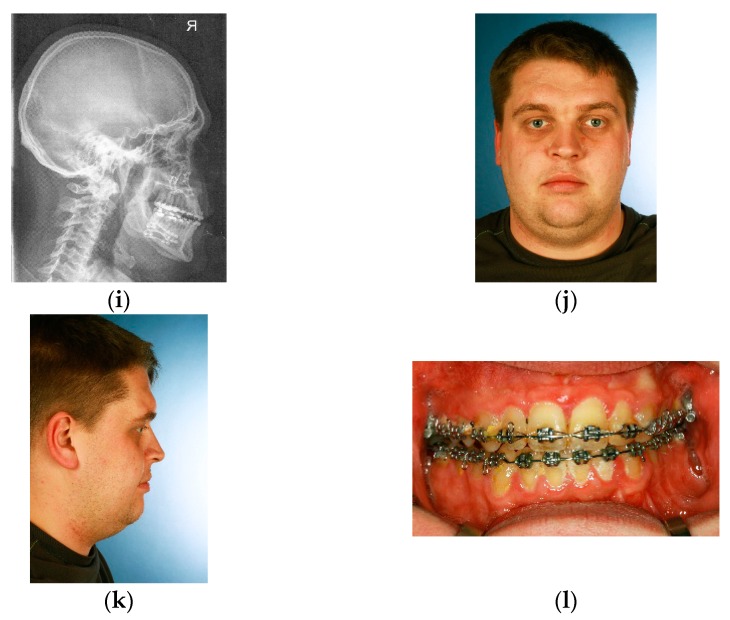
(**a**–**d**) Preoperative clinical and radiological situation in a 25 year-old patient affected by hemimandibular hyperplasia; (**e**) Corresponding scintigraphy demonstrating increased tracer uptake in the right mandibular condyle; (**f**) Specimen after surgical resection of the “active condyle”; (**g**,**h**) Surgical management of the transverse maxillary deficiency by transpalatal distraction; (**g**) during orthodontic alignment; (**h**) palatal view after device removal; (**i**–**l**) Radiologic and clinical situation after combined treatment—1: simultaneous condylectomy & transpalatal distraction; 2: orthodontic alignment; 3: reorientation of the mandibulomaxillary complex by “two-jaw-surgery”.

A 25-year-old male patient with obvious dentofacial deformity due to an underlying unilateral right hemimandibular hyperplasia (Figure 1a–d). Preoperatively an increased tracer uptake in the scintigraphy demonstrates an “active condyle,” which is likely responsible for persistent mandibular growth (Figure 1e). The initial surgical intervention consisted of a high condylectomy in combination with the insertion of a transpalatal distraction device in order to correct the transverse maxillary deficiency and prevent further mandibular growth (Figure 1f,g). After the orthodontic closure of the diastema (Figure 1h) and levelling of the dental arches, surgical correction was subsequently performed by “two-jaw surgery”: the maxillary advancement after LeFort I osteotomy and mandibular setback after a bisagittal split osteotomy (BSSO) resulted in a clockwise rotation of the occlusal plane and improved jaw relations (Figure 1i). Clinical situation after material removal and with ongoing orthodontic treatment (Figure 1j–l).

### Case 2 (Figure 2a–i)

**Figure 2:**
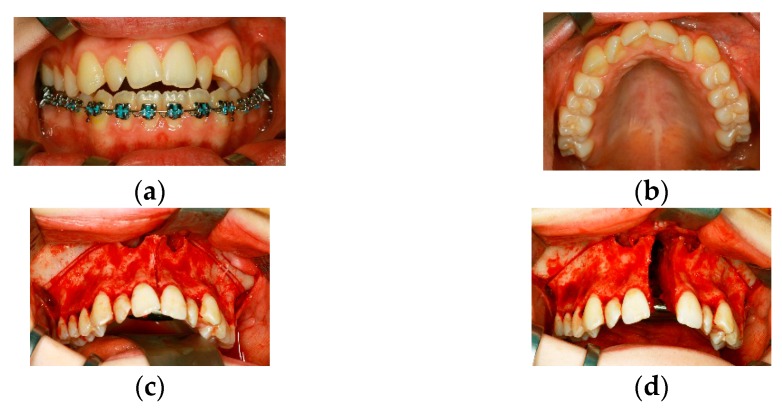

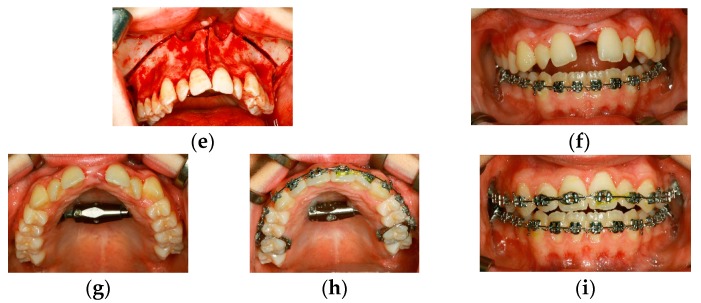
(**a**,**b**) Preoperative clinical situation in a 15-year old female patient affected by transverse maxillary deficiency and dental crowding in the anterior maxillary arch; (**c**–**e**) Intraoperative situation during insertion of a transpalatal distraction device, (**c**) anterior maxillary osteotomies were performed by piezosurgery, (**d**) intraoperative activation of the device to the extent needed, for that purpose bone strips have to be removed bilaterally (**e**); (**f**–**i**) Clinical situation during active distraction (**f**,**g**) and after orthodontic closure of the diastema (**h**,**i**)—residual open bite is likely to be managed by orthodontic means alone.

A 15-year-old female patient with an open bite and a transversal maxillary deficiency, predominantly in the anterior maxillary arch (Figure 2a,b). Surgery consisted in a LeFort I osteotomy modified according to Betts without the mobilization of the pterygomaxillary junction [19] and the insertion of a Surgitec transpalatal “All-in-one”-type distraction device. Osteotomies were performed by piezosurgery. Sufficient removal of bone is required in order to guarantee a stress-relieved expansion of both maxillary segments up to a diastema width of 10 mm; the parallel movements of the bony segments can be controlled by intraoperative activation of the device (Figure 2c–e). With regard to the clinical situation after the end of active distraction, the width of diastema was achieved as scheduled (10 mm, Figure 2f,g). Nine months after transpalatal distraction, the orthodontic treatment is almost finished, and the residual open bite is likely to be managed by orthodontic means alone (Figure 2h,i).

### Case 3 (Figure 3a–h)

**Figure 3:**
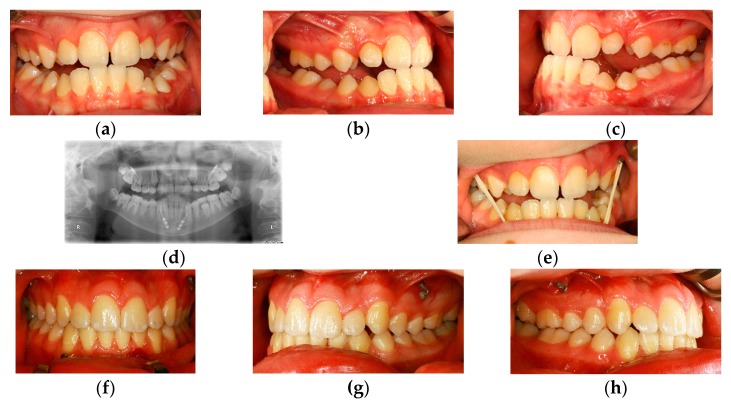
(**a**–**c**) Clinical situation in an eleven-year old boy demonstrating a class III malocclusion during late mixed dentition; (**d**,**e**) Radiologic (**d**) and clinical situation after insertion of skeletal anchorage devices according to H. de Clerck; class III elastics are inserted in order to support maxillary growth (**e**); (**f**–**h**) Clinical situation after “four years of class III elastics” demonstrating correct occlusal relations.

The occlusal situation in an 11-year-old male patient before puberty affected by class III malocclusion (Figure 3a–c): the orthopantomography and intraoral situation after the insertion of the skeletal anchorage. According to the concept of H. de Clerck, class III elastics were applied to these elements in order to support maxillary growth (Figure 3d,e). The clinical situation after four years demonstrating physiologic overjet and overbite, which is likely due to the skeletal effect of the class III elastics (Figure 3f–h).

### Case 4 (Figure 4a–j)

**Figure 4:**
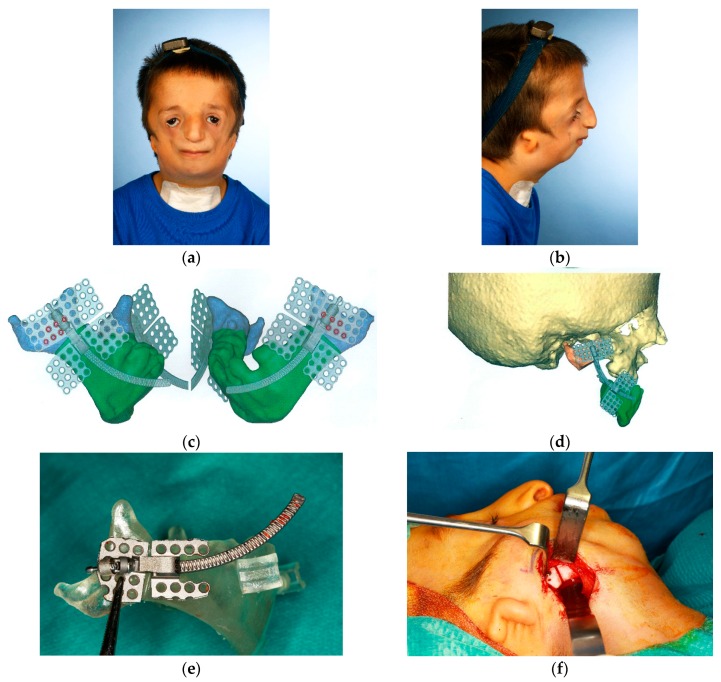

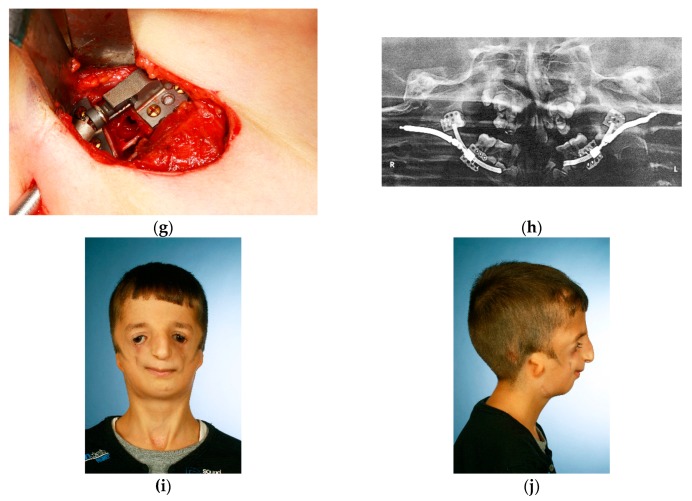
(**a**,**b**) Preoperative situation in a seven- year old boy affected by Treacher-Collins-Syndrome, due to airway impairment tracheostomy was present since the first year of life; (**c**,**d**) Virtual planning of mandibular reconstruction by curvilinear distraction; (**e**–**g**) Intraoperative situations; (**e**) distraction device adapted to the mandible, (**f**) corresponding cutting guides in place for piezo-assisted osteotomy, (**g**) intraoperative activation of the distraction device demonstrating the intact neurovascular bundle; (**h**) Radiological situation at the end of distraction (bilateral activation of 20 mm); (**i**,**j**) Clinical situation one year after computer-assisted lower facial reconstruction by bilateral curvilinear mandibular distraction.

The preoperative situation in a seven-year-old boy affected by Treacher-Collins-Syndrome. Due to airway impairment tracheostomy was present since his first year of life (Figure 4a,b). Computer-assisted planning of the lower facial reconstruction was carried out by means of bilateral internal curvilinear distraction devices (DePuySynthes Curvilinear distraction device 1.3 mm). Planning by “TruMatch^®^” based on CT-derived DICOM data sets were processed by DePuy Synthes in cooperation with Materialise (Leuven, Belgium) (Figure 4c,d). The preoperative adaptation of the distraction device according to the virtual planning and patient specific mandibular model are shown (Figure 4e). The intraoperative situation with a cutting guide placed at the right mandibular angle for correct transfer of the planning (Figure 4f). The intraoperative situation after piezo-assisted mandibular osteotomy; the placement and activation of the distraction device demonstrating a preserved inferior alveolar nerve (Figure 4g). An orthopantomogramm during the active bilateral curvilinear mandibular distraction period (Figure 4h). The clinical situation one year after the computer-assisted lower facial reconstruction (Figure 4i,j). The decanulation and tracheal reconstruction was performed simultaneously with device removal six months after the end of active distraction without any airway impairment. Further corrective surgery of ears, eyelids and zygomatic regions is postponed in accordance with patient’s decision.

## 4. Discussion

Today, computer-assisted technologies are well established in all fields of surgery. The benefit of these technologies for orthognathic surgery has been emphasized by different authors [20,21,22,23]. In particular, when using a “surgery first” approach—which had already been proposed 25 years ago [24]—computer-assisted 3D-planning is supportive [25]. The main advantage of this concept is the early visible effect for the patient as well as an obvious shortening of overall treatment time due to the accelerated orthodontic movements after surgery [26,27,28]. However, at the moment there is no evidence that the results are superior when compared to the classic approach with pre- and postsurgical orthodontic treatment. It is emphasized that careful patient selection as well as very close cooperation between surgeon and orthodontist are mandatory in order to avoid treatment failure [29].

Computer-assisted technologies are likely to gain more and more importance in the planning and transfer of dentofacial corrections. Integrated digital workflows have been transferred to the whole craniomaxillofacial framework and have gained acceptance in craniofacial, microvascular and transplant surgery [30,31,32]. The DICOM data-based fabrication of patient-specific wafers/splints/cutting guides for dental implantology and/or orthognathic corrections is well established nowadays and provides an exact transfer of the preoperative planning, as demonstrated in patient 4. These technologies also have an impact on quality control: through image fusion or the superimposition of pre- and postoperative datasets, effective skeletal changes can be documented and quantified. Surgical techniques may consequently be improved or adapted by 3D-evaluation or may help in the planning of secondary corrections. The additional implementation of datasets of surface scans and photos, as well as the use of navigational devices and the fabrication of patient-specific implants, might further contribute to individualized surgical approaches in the interdisciplinary correction of dentofacial anomalies [33,34]. Although surgical documentation, patient education and teaching are also improved by computer-assisted workflows, some drawbacks must certainly be resolved; currently, these technologies require additional time, costs and effort and are not available everywhere [11]. In our own series they were only used in selected patient cases. With respect to irradiation exposure and the ALARA principle, it is arguable whether the straightforward correction of a conventional dentofacial anomaly justifies extended imaging procedures [35].

According to our results, transpalatal distraction—initially described by Mommaerts in 1999 [36]—can be regarded as state-of-the-art for maxillary expansion if conservative attempts have failed. It allows for the individualized correction of a present transverse maxillary deficiency and may be indicated already before skeletal maturity, especially in syndromal or dysostotic conditions [37]. In spite of the fact that, according to Nada’s web-based survey distraction osteogenesis cannot yet be regarded as evidence-based care [38], the principle of gradual expansion of bone and surrounding soft tissues as realized by distraction osteogenesis seems to provide reliable results in patients affected by dentofacial and craniofacial anomalies [39,40]. Skeletal anchorage also offers new perspectives for the early correction of dentofacial anomalies. H. de Clerck was the first to point out that there is a good option to influence midfacial growth through the use of skeletally anchored class III elastics, possibly avoiding subsequent orthognathic surgery [18] (Figure 3). Initial results are very promising, however, for the moment there are no long-term results which provide clear evidence for this theory [41].

The contemporary approach for the planning, transfer and evaluation of complex surgical procedures for the skeletal correction of dentofacial anomalies can be summarized as follows:

1. Preoperative imaging and acquisition of DICOM datasets;
2. Processing of datasets and generation of .stl-files for the fabrication of patient-specific models or further virtual planning;
3. Virtual planning of orthognathic surgery and virtual design of cutting guides/splints/wafers/patient-specific implants, if needed;
4. Surgical correction, integration of navigational systems, if needed;
5. Postoperative imaging, superimposition of pre- and postoperative datasets, evaluation of skeletal changes.

## 5. Conclusions

Technical innovations influence the interdisciplinary treatment approach of patients affected by dentofacial anomalies. The planning, transfer, evaluation and documentation of orthognathic procedures can be supported by computer-assisted technologies. Whether the treatment results are truly superior when compared to conventional methods remains open and must be evaluated further. Despite integrated workflows, it cannot be guaranteed that the final result will be in accordance with a patient’s expectations. A patient-specific approach based on confidence and compliance, as well as on solid craftsmanship, will remain the key factors in order to provide a satisfying outcome for all participants of this interdisciplinary team approach.

## Figures and Tables

**Table 1:** Distribution of orthognathic procedures from January 2007 up to May 2015.

Year	BSSO	LeFort I	TJS	DO	Other	Overall
2007	9	9	7	10	3	**38**
2008	11	8	8	3	4	**34**
2009	10	5	5	4	3	**27**
2010	4	5	6	4	2	**21**
2011	14	10	11	15	1	**51**
2012	4	8	9	13	14	**48**
2013	7	17	3	22	3	**52**
2014	11	13	4	15	9	**52**
5-2015	6	6	7	7	1	**27**
**Overall**	**76**	**81**	**59**	**93**	**40**	**350**

BSSO: Bisagittal Split Osteotomy; TJS: Two-Jaw-Surgery; DO: Distraction Osteogenesis.
